# MRI as a Tool to Assess Interstitial Cystitis Associated Bladder and Brain Pathologies

**DOI:** 10.3390/diagnostics11122298

**Published:** 2021-12-08

**Authors:** Rheal A. Towner, Nataliya Smith, Debra Saunders, Robert E. Hurst

**Affiliations:** 1 Advanced Magnetic Resonance Center, Oklahoma Medical Research Foundation, Oklahoma, OK 73104, USA; Nataliya-Smith@omrf.org (N.S.); Debra-Saunders@omrf.org (D.S.); 2 Department of Urology, University of Oklahoma Health Sciences Center, Oklahoma, OK 73104, USA; reh123@cox.net

**Keywords:** interstitial cystitis/bladder pain syndrome (IC/BPS), magnetic resonance imaging (MRI), preclinical, clinical

## Abstract

Interstitial cystitis/bladder pain syndrome (IC/BPS) is a chronic, often incapacitating condition characterized by pain seeming to originate in the bladder in conjunction with lower urinary tract symptoms of frequency and urgency, and consists of a wide range of clinical phenotypes with diverse etiologies. There are currently no diagnostic tests for IC/BPS. Magnetic resonance imaging (MRI) is a relatively new tool to assess IC/BPS. There are several methodologies that can be applied to assess either bladder wall or brain-associated alterations in tissue morphology and/or pain. IC/BPS is commonly associated with bladder wall hyperpermeability (BWH), particularly in severe cases. Our group developed a contrast-enhanced magnetic resonance imaging (CE-MRI) approach to assess BWH in preclinical models for IC/BPS, as well as for a pilot study for IC/BPS patients. We have also used the CE-MRI approach to assess possible therapies to alleviate the BWH in preclinical models for IC/BPS, which will hopefully pave the way for future clinical trials. In addition, we have used molecular-targeted MRI (mt-MRI) to quantitatively assess BWH biomarkers. Biomarkers, such as claudin-2, may be important to assess and determine the severity of BWH, as well as to assess therapeutic efficacy. Others have also used other MRI approaches to assess the bladder wall structural alterations with diffusion-weighted imaging (DWI), by measuring changes in the apparent diffusion coefficient (ADC), diffusion tensor imaging (DTI), as well as using functional MRI (fMRI) to assess pain and morphological MRI or DWI to assess anatomical or structural changes in the brains of patients with IC/BPS. It would be beneficial if MRI-based diagnostic tests could be routinely used for these patients and possibly used to assess potential therapeutics.

## 1. Diagnostic Criteria for IC/BPS?

Interstitial cystitis/bladder pain syndrome (IC/BPS) is a chronic, often incapacitating condition characterized by pain seeming to originate in the bladder in conjunction with lower urinary tract symptoms of frequency and urgency, and consists of a wide range of clinical phenotypes with diverse etiologies [1,2]. Historically, the diagnostic criteria for IC/BPS have varied over time [3]. Originally, it was a diagnosis of exclusion; after exclusion of other causes, such as bladder cancer or carcinoma in situ, patients with pain, urgency, and frequency were given the diagnosis of IC/BPS. These criteria led to considerable confusion in research studies because “pain, urgency, and frequency” were ill-defined. In addition, as a result, evaluating therapies was difficult due to the variability in diagnostic criteria, which resulted in heterogeneity in the patient population. The National Institutes of Health (NIH) supported a multicenter research project, the Interstitial Cystitis Database (ICDB), to test 44 diagnostic criteria [4], which led to a more restrictive set of diagnostic criteria intended to standardize research studies. These criteria required cystoscopy and biopsy with hydrodistension to test for petechial bleeding and identification of characteristic morphologic changes in the urothelium [4]. One unintended result was that these restrictive criteria intended only to improve research studies became de facto diagnostic criteria in clinical practice [5].

The ICDB demonstrated that not only were the NIH research criteria too restrictive, they were not specific either. This information led to a loosening of criteria and dropping a biopsy requirement [6]. Even cystoscopy was no longer mandatory. Nonetheless, this broadened definition led to a more heterogeneous patient population with likely inclusion of multiple etiologies, further contributing to difficulty in clinical trials and targeting therapy [7]. However, current clinicopathological and genomic data propose that IC/BPS should be subclassified by the presence or absence of Hunner’s lesions or ulcers, on top of the basic diagnosis that is established by clinical phenotyping based on symptomatology with standardized symptom score vehicles [8,9]. This leaves a wide range of patients in the non-Hunner’s lesion category [1,10], and considerable evidence suggests IC/BPS may represent a heterogeneous syndrome [11].

IC/BPS is often underdiagnosed and mistreated [12]. The complications in diagnosis stem from the lack of a clear etiology. In 1991, Parsons demonstrated that IC/BPS patients showed significantly increased permeability to urea instilled into the bladder of IC patients as compared to controls (25% vs. 5%) [13]. Parsons then proposed on the basis of these findings that IC/BPS involved epithelial dysfunction and loss of bladder; the epithelial surface glycosaminoglycan layer that Parsons proposed acts as a permeability barrier [14]. However, the theory has been controversial, possibly because no mechanism has emerged to explain how the urothelium becomes dysfunctional, and other surface molecules, such as tight junction proteins [15] and uroplakins [16], were also demonstrated to function as permeability barriers. The evidence, however, is conclusive that the urothelium is dysfunctional [13,17]. Histopathologic examination of biopsies by several investigators has identified an apparently altered differentiation program with morphologic and molecular differences in protein expression from normal [18,19,20]. The normal polarity of the urothelium can be lost with the disappearance of the apical umbrella cells with their components of the permeability barrier, such as tight junctions, uroplakins, and proteoglycans, and alterations in the expression of differentiation markers, such as KRT 18 and KRT20 [18,19,21,22] and others [23]. In a study of urothelial morphology and protein expression in single patient biopsies, the overall pattern varied from nearly normal to highly altered. A later study of multiple sections of bladders from cats with Feline Interstitial Cystitis (FIC) showed the abnormalities were virtually identical to what is found in humans, but the availability of full sections showed the changes were focal in cats [18]. This observation suggested the findings of a normal or nearly normal urothelium in human patients could represent failure to sample an appropriate area, or alternately could represent a heterogeneity in the disease such that not all patients exhibit urothelial dysfunction. The etiology is unknown, and given the variable symptoms and widespread effects on the lower abdomen, such as a high comorbidity with irritable bowel syndrome (IBS), dissecting cause from effect has been difficult and generally unsuccessful.

Nonetheless, a crucial theme has been an association with increased permeability of the bladder wall [24,25]. The urothelial GAG layer has a crucial role in delivering a permeability barrier to prevent penetration of urinary toxins and pathogens into the bladder wall [24,26]. In multiple animal models [27,28,29] as well as in cell culture models [30], disruption of the GAG layer results in an induction of permeability that can be restored to normal impermeability by treatment with GAGs [31,32,33]. Most convincing is that digestion of chondroitin sulfate on the bladder surface with the specific enzyme chondroitinase ABC alone can induce the same degree of permeability as treatment with protamine sulfate [33]. Given the disruption of the GAG layer seen in biopsies, increased permeability is thought to contribute to the development of IC/BPS [24,26]. Data support that the replacement of GAGs can restore the GAG layer in IC/BPS, resulting in the reduction of pain, inflammation, and other symptoms in some patients [24,26]. However, restoring bladder impermeability may not be sufficient for treatment of IC/BPS. Response rates for intravesical GAG therapy are less than optimal; whether this is due to the therapy not being optimized (i.e., impermeability is only maintained intermittently) or the symptoms arise from a systemic disease rather than only locally in the bladder is very unclear. IC/BPS patients suffer from a number of comorbidities at significantly higher rates than do non-IC/BPS patients, which suggests that IC/BPS could represent a systemic problem [34,35].

We showed that inducing colitis in a rat model would not only increase bowel permeability, but the permeability of the bladder increased as well, and, likewise, inducing bladder permeability increased bowel permeability [29]. This result clearly demonstrates visceral organ crosstalk and provides a model for investigating the mechanisms that lead to the high comorbidity of lower pelvic pain syndromes. It is also possible that with time and continued pain originating from the bladder and organ crosstalk that CNS remodeling could result in a chronic pain syndrome in which the stimulus of bladder pain resulting from increased permeability is no longer required for the perception of pain by the patient [36,37]. Such patients might show limited or even no response to intravesical GAG therapy or might show only a delayed response.

Whether the altered changes in the urothelium that increase permeability in IC are primary or the secondary result of other processes, intravesical GAG therapy can at least sometimes reduce the symptoms of pain, urgency, and frequency. It seems the management of IC/BPS could be greatly simplified by having a minimally invasive test for IC/BPS that does not depend upon cystoscopy and biopsy but could at least detect increased bladder permeability. Even better would be to be able to identify CNS remodeling as well because such patients likely would need additional therapeutic interventions beyond intravesical GAG therapy. Given the advances in imaging with MRI that have occurred in recent years, MRI has the potential for directly imaging bladder wall permeability, as well as brain alterations associated with pain and morphology, and perhaps even CNS remodeling. In this communication, we review recent studies conducted by ourselves and others who used MRI imaging for the diagnosis of IC/BPS. Our group developed a contrast-enhanced magnetic resonance imaging (CE-MRI) approach to assess BWH in preclinical models for IC/BPS, as well as for a pilot study for IC/BPS patients. We also used the CE-MRI approach to assess possible therapies to alleviate the BWH in preclinical models for IC/BPS, which will hopefully pave the way for future clinical trials. In addition, we used molecular-targeted MRI (mt-MRI) to quantitatively assess BWH biomarkers. Others have also used other MRI approaches to assess the bladder wall structural alterations with diffusion-weighted MRI or diffusion-weighted imaging (DWI) by measuring changes in the apparent diffusion coefficient (ADC), diffusion tensor imaging (DTI) as an extension of DWI, as well as using functional MRI (fMRI) to assess pain, and morphological MRI or DWI to assess anatomical or structural changes in the brains of patients with IC/BPS. This review will discuss the various uses of MRI that can be helpful in the diagnosis of IC/BPS-associated bladder and brain pathologies in both preclinical and clinical studies.

## 2. MRI Imaging

MRI, in its most widely used scenario, is essentially a water distribution image that has contrasting effects in different tissues due to the presence of water hydrogen protons and two relaxation time constants (T1 or spin-lattice/longitudinal relaxation and T2 or spin-spin/transverse relaxation). These three characteristics are endogenous to the tissue being investigated. With the use of exogeneous contrast agents, T1 and/or T2 relaxation times can be regionally changed. Gadolinium (Gd)-based contrast agents will increase MRI signal intensities, as well as shorten T1 values, whereas iron oxide contrast agents decrease MRI signal intensities and shorten T2 values. In essence, this is what is referred to as CE-MRI. Particular MRI sequences are used to manipulate the contrast-enhancement effect. Contrast agents are usually excluded from impermeable membranes, such as the bladder wall, however, if there is damage, then the contrast agent can provide information regarding the tissue damage. Molecular-targeted MRI (mt-MRI) can also be an extension of CE-MRI that allows direct measurement of the expression levels/changes associated with specific biomarkers. Again, the tissue in question must be able to allow distribution of the mt-MRI agents or be damaged to allow access. mt-MRI probes are used which contain a targeting ligand (e.g., antibody or peptide) and a signaling component (Gd- or iron oxide-based contrast agents). MRI also has the ability to measure biophysical/biomolecular changes in cerebral blood flow (as measured with perfusion imaging), blood-oxygen level-dependent (BOLD) (as measured using fMRI), and the apparent diffusion coefficient (ADC) (as measured by DWI). An extension of DWI is diffusion tensor imaging, which measures alterations in the white matter fiber tract. All of these methods will be further discussed with specific examples below. MR spectroscopy can also be conducted on several MRI scanners, allowing the measurement of several metabolites (either hydrogen-, phosphorous-, fluorine-, or carbon-13-containing molecules), however this is beyond the scope of this review.

### 2.1. Using CE-MRI to Assess BWH in Preclinical Rodent Models and Clinically

**Figure 1:**
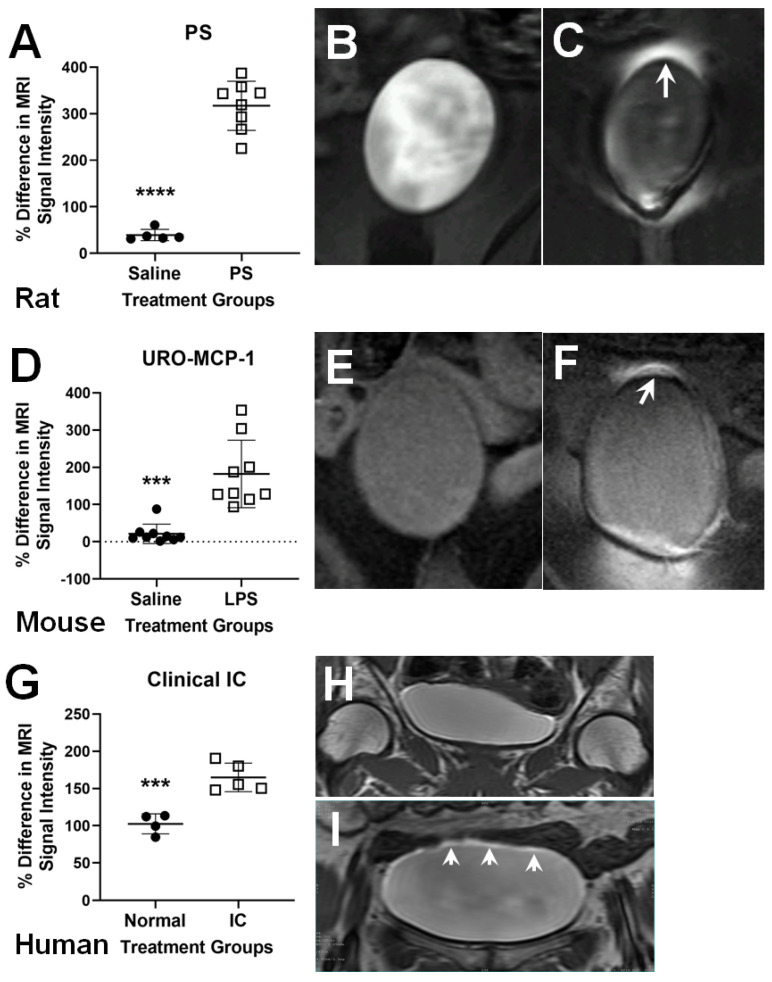
Bladder wall hyperpermeability CE-MRI in preclinical models and human IC/BPS. Rat protamine sulfate (PS)-induced BWH model (**A**–**C**). (**A**) Quantitative assessment of BWH in a rat PS-induced cystitis model treated with either saline (normal control; closed circles) or PS (open squares); 3 days post PS. There was a significant difference between the saline control and PS groups (**** *p* < 0.0001). (**B**,**C**) Representative CE-MR images of saline- (**B**) or PS- (**C**) rat bladders. White arrow in panel “C” depicts high MRI signal intensity (SI) due to BWH. Transgenic mouse IC/BPS model (**D**–**F**). (**D**) Quantitative assessment of BWH in a transgenic URO-MCP-1 mouse lipopolysaccharide (LPS)-induced IC model treated with either saline (normal control; closed circles) or LPS (open squares); 5 days post LPS. There was a significant difference between the saline and LPS groups (*** *p* < 0.001). (**E**,**F**) Representative CE-MR images of saline- (**E**) or LPS- (**F**) mouse bladders. White arrow in panel “F” depicts high MRI signal intensity (SI) due to BWH. Clinical IC/BPS patients (**G**–**I**). (**G**) Quantitative assessment of BWH in normal controls (closed circles) or IC patients (open squares). There was a significant difference between normal and IC groups (*** *p* < 0.001). (**H**,**I**) Representative CE-MR images of normal and IC patients. White arrow in panel “I” depicts high MRI signal intensity (SI) in bladder wall due to BWH. Figures were modified from those published in Refs. [28,38,39].

We initially used CE-MRI in a rat BWH model where intravesical-administered protamine sulfate (PS) was used cause structural alteration of the bladder wall [28]. MRI signal intensities (SIs) were found to increase in the surrounding bladder wall, particularly in the dome region, as a result of the increased leakage of the MRI contrast agent, Gd-DTPA (gadolinium- diethylenetriaminepentaacetic acid), administered intravesically (see Figure 1A–C where there was a significant increase in the percent (%) difference in MRI signal intensity (SI) for the PS group (*p* < 0.0001) due to the leakage of Gd-DTPA as shown in Figure 1A) [28]. Gd-DTPA normally does not go through an impermeable bladder wall. Of interest, we were also able to detect bladder-colon crosstalk associated colitis via intravenous (iv) administration of Gd-DTPA [28], as well as BWH from colon-bladder crosstalk following the induction of intestinal cystitis with trinitrobenzene sulfonic acid (TNBS) [38]. CE-MRI of BWH could therefore also be applied to assess colon–bladder crosstalk in inflammatory bowel disease (IBD) patients, as well as other inflammatory colon diseases. Additionally, we were able to demonstrate an increase in MRI SIs in the regions surrounding the dome of the bladder wall in a transgenic URO-MCP-1 mouse model for IC/BPS following intravesical lipopolysaccharide (LPS) exposure (see Figure 1D–F where there was a significant increase in the % difference in MRI SI in the LPS group (*p* < 0.001) due to leakage of Gd-DTPA) [39]. A similar CE-MRI approach to assess urothelial regeneration in rat bladders, which were augmented with permeable porcine small intestinal submucosa, was also used [40], indicating that this methodology can be expanded to other bladder conditions. Following the preclinical rat PS-induced BWH study, we then conducted a human pilot study comparing IC/BPS patients with normal individuals and clearly demonstrated the clinical feasibility of detecting BWH in IC/BPS [41]. Due to the increased thickness of the human bladder wall, BWH was detected within the bladder wall (see Figure 1G–I where there was a significant increase in % difference in MRI SI for the IC bladders (*p* < 0.001) compared to controls) [41] rather than the outside regions as seen in the preclinical models [28,38,39]. Another group used a mixture of both Gd-DTPA (Gadavist) and an iron oxide contrast agent, ferumoxytol, to further enhance bladder wall contrast in rats with induced cystitis (PS-induced) [42] and more recently in patients with IC [43].

**Figure 2:**
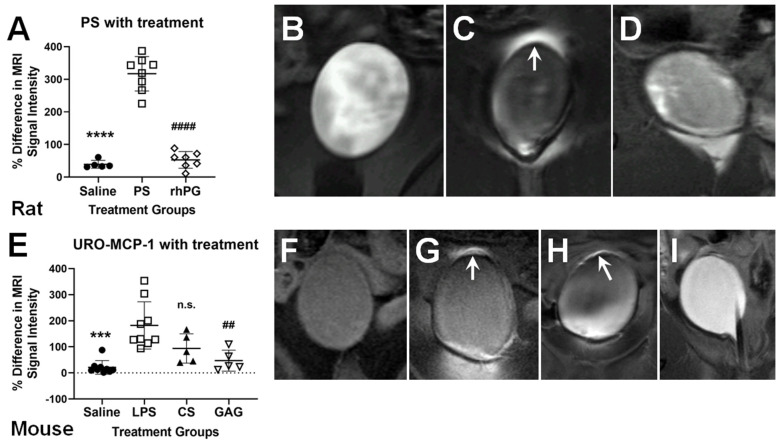
Using CE-MRI to detect therapeutic efficacy against BWH. Rat protamine sulfate (PS)-induced BWH model with treatment (**A**–**D**). (**A**) Quantitative assessment of BWH in a rat protamine sulfate PS-induced cystitis model treated with either saline (normal control; closed circles)), PS (open squares) or a recombinant human proteoglycan (rhPG; open diamonds); 3 days post PS. There was a significant difference between the rhPG and PS groups (#### *p* < 0.0001). **** *p* < 0.0001 (PS-treated significantly higher than saline control). (**B**–**D**) Representative CE-MR images of saline- (**B**), PS- (**C**), or rhPG-treated (**D**) rat bladders. White arrow in panel “C” depicts high MRI signal intensity (SI) due to BWH. Transgenic mouse IC/BPS model with treatment (**E**–**I**). (**E**) Quantitative assessment of BWH in a transgenic URO-MCP-1 mouse lipopolysaccharide (LPS)-induced IC model treated with either saline (normal control; closed circles), LPS (open squares), chondroitin sulfate (CS; closed upward triangles)), or a super-glycosaminoglycan (GAG; open downward triangles); 5 days post LPS. There was a significant difference between the GAG and LPS groups (## *p* < 0.01). *** *p* < 0.001 (PS-treated significantly higher than saline control). The CS group was not significantly different than the LPS group. (**F**–**I**) Representative CE-MR images of saline- (**F**), LPS- (**G**), CS- (**H**) or GAG-treated (**I**) mouse bladders. Note BWH (white arrows) in frames (**G**,**H**). Figures were modified from those published in Refs. [44,45].

Regarding therapeutic interventions, we were able to use CE-MRI to demonstrate recently that if a recombinant human proteoglycan (see Figure 2A,D) [44] or a high molecular-weight GAG (see Figure 2E,I) [45] biopolymers are used, that BWH is significantly decreased, as measured with decreasing MRI SIs compared to PS- or LPS-induced rodents, respectively, with increased hyperpermeabilities.

### 2.2. Using mt-MRI to Assess BWH Biomarkers

mt-MRI using Gd-based MRI contrast agents with antibodies attached to an albumin linker (antibody-albumin-Gd-DT.

**Figure 3:**
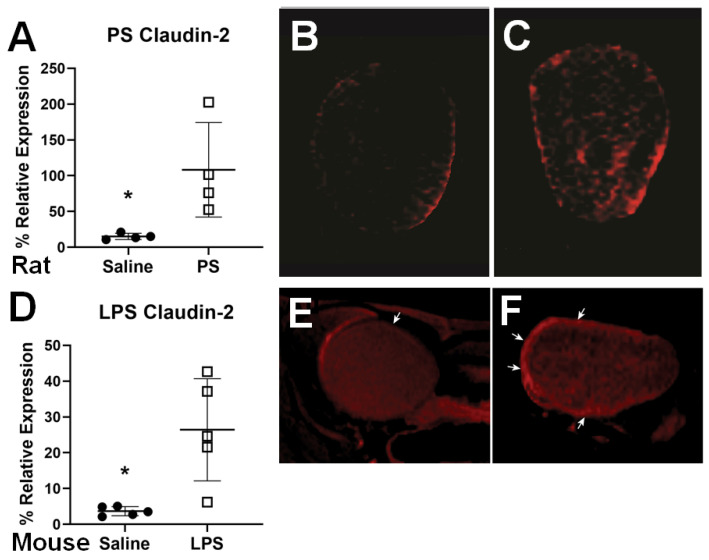
Molecular-targeted MRI (Mt-MRI) of claudin-2 in a rat PS-induced cystitis model (**A**–**C**) or URO-MCP-1 transgenic mouse model (**D**–**F**). (**A**) Quantitative assessment of claudin-2 probe expression in a rat PS-induced cystitis model. There was a significant difference between saline (closed circles) and PS groups (open squares) (* *p* < 0.05). Representative mt-MR images in saline- (**B**) or PS-treated (**C**) rat bladders administered with a claudin-2 Gd-based probe. Note high expression of claudin-2 in frame C. (**D**) Quantitative assessment of claudin-2 probe expression in a mouse URO-MCP-1 LPS-induced IC model. There was a significant difference between saline (closed circles) and LPS groups (open squares) (* *p* < 0.05). Representative mt-MR images in saline- (**E**) or LPS-treated (**F**) mouse bladders administered with a claudin-2 Gd-based probe. Note high expression of claudin-2 in frame R (white arrows). Figures were modified from those published in Refs. [39,46].

PA-biotin) were used by our group to assess BWH biomarkers, such as decorin, vascular endothelial growth factor receptor 1 (VEGFR1) claudin-2 in a rat PS-induced BWH model (see Figure 3A–C where the claudin expression is significantly increased in the PS group (*p* < 0.05)) [46], and claudin-2 in the transgenic URO-MCP-1 mouse model for IC/BPS (see Figure 3D–F where the claudin expression is significantly increased in the LPS group (*p* < 0.05)) [39]. Contrast difference images indicate either the increased levels of claudin-2 [39,46] or a decrease in the levels of either decorin or VEGFR1 [46]. Decreased protein expression for decorin was previously found in feline interstitial cystitis bladders [18]. It was also found that the expression of VEGFR1 is substantially downregulated in interstitial cystitis (IC) compared to controls in humans [47]. Conversely, claudin-2 (Cldn2) is a tight junction-associated protein, which has been found to be upregulated in bladder biopsies from patients with IC/BPS, as well as in rodent models for cystitis [48,49]. The mt-MRI approach can be used in future studies to assess whether therapies may be effective against BWH biomarkers.

### 2.3. Using DWI to Assess Bladder Wall Structural Damage

Recently, diffusion-weighted MRI (DW-MRI) or DWI has been used for the diagnosis of IC/BPS, where a high DW-MRI signal was found to be substantially higher in IC patients compared to normal controls [50,51]. They suggested that having a positive DW-MRI signal was suggestive of IC [50]. Unfortunately, in either study, they did not specifically measure the apparent diffusion coefficient (ADC), which should increase with increased inflammation, however, which may also contribute to calculation errors if low b-values are used which also takes into consideration the effect of perfusion [52].

Quantification and correction of distortion for DW-MRI (or DWI) were assessed in a muscle-invasive bladder cancer plantom recently [53], which could be applied to measure geometric distortion in IC patient bladder walls as well. In addition to DWI, diffusion kurtosis imaging (DKI) was used to differentiate between muscle-invasive BC and non-muscle-invasive BC [54]. DKI is believed to better reflect the deviation from a Gaussian distribution due to the irregularity and heterogeneity of cell microstructure and tissue components [54], which could also be applied to assess BWH-related structural alterations. In our clinical pilot study assessing CE-MRI with BWH, we applied kurtosis (shape of probability distribution) and skewness (measure of probability distribution asymmetry) in association with contrast enhancement [41], but this could be extended to DWI as well.

### 2.4. Using DTI (Diffusion Tensor Imaging) to Assess IC/BPS-Associated Pain

Diffusion tensor imaging (DTI), a specific DWI technique that fits a mathematical tensor to diffusion MR measurements obtained in various orientations, was used to show that patients with urological chronic pelvic pain syndrome (UCPPS), which includes IC/BPS, have extensive microstructural differences within the brain that appear to be localized to regions associated with the perception and integration of sensory information and pain modulation and overall seem to be a consequence of longstanding pain [55]. More specifically, a brainstem region was identified that showed a strong correlation between both ADC (apparent diffusion coefficients) and FA (fractional anisotropy) with urinary MMP9 (matrix metalloproteinase 9) levels, as well as a correlation between both ADC and FA and the urinary MMP9/NGAL complex [55]. In addition, the results also identified significant correlations between FA and urinary MMP9 in white matter adjacent to sensorimotor regions, as well as a correlation in similar sensorimotor regions when examining ADC and urinary MMP2 levels as well as FA and the urinary MMP9/NGAL complex [55]. A large, diffuse cluster of white matter was also identified as having a strong correlation between both ADC and FA with urinary NGAL levels [55]. This approach could be used in the future to access specific regions of CNS remodeling that could be used to pinpoint areas of interest to monitor when evaluating potential therapeutics.

### 2.5. fMRI Investigations in Brain Pain Associated with IC/BPS

The pathophysiology of IC/BPS is believed to involve central disturbance in the processing of pain and viscerosensory signals [56]. Specifically, it was shown that there were altered frequency distributions in viscerosensory (postinsula), somatosensory (postcentral gyrus), and motor regions (anterior paracentral lobule, and medial and ventral supplementary motor areas) and that the anterior paracentral lobule, and medial and ventral supplementary motor areas showed increased functional connectivity to the midbrain (red nucleus) and cerebellum, particularly when pain was reported during bladder filing [56]. This study suggested that patients with IC/BPS have a sensorimotor element to the pathological condition involving an alteration in intrinsic oscillations and connectivity in a cortico-cerebellar network previously related with bladder function [56].

The remodeling of functional neuronal connectivity in chronic widespread pain (CWP) patients from brain networks related to chronic pain for changes related to pain sensitivity, psychological strain, and experienced pain was recently assessed using fMRI [57]. More specifically, CWP patients showed decreased connectivity in the inferior posterior cingulate cortex (PCC) in the default mode network (DMN) DMN and increased connectivity in the left anterior insula/superior temporal gyrus in the salience network (SN) when compared to controls [57]. Moreover, higher pain sensitivity in CWP, when compared to controls, was related to increased connectivity within the SN (between the left and right insula) and between the SN and DMN (between the right insula and left lateral parietal cortex) [57]. A similar approach could also be applied to IC/BPS patients, as well as establishing the effectiveness of therapies that could target either the brain and/or the bladder. Of interest, resting state fMRI (rs-fMRI) was recently used to detect brain function changes across the micturition loci, including subregions of the salience, sensimotor, and default networks in response to bladder filling (drinking of water) and urinary urge [58]. Arterial spin label fMRI was also used to measure an increase in regional cerebral blood flow following bladder distension in IC patients, particularly the supplemental motor area, the motor and sensory cortex, the insula, the hippocampal structures, and the middle and posterior cingulate areas compared with controls [59].

### 2.6. Other MRI Studies Either Associated with IC/BPS Or That Could Be Applied for IC/BPS

Voxel-based MRI morphometry was used to determine whether IC patients had changes in brain morphology compared to normal individuals [60]. It was found that IC patients had substantially increased gray matter volumes in the right primary somatosensory cortex, which is associated with greater pain, mood/anxiety, and urological symptoms [60].

MRI was previously used to assess pelvic floor hypertonicity by measuring alterations in levator muscles (shortened), the posterior puborectalis angle (wider), the H line (shorter), and vaginal cuff and bladder neck distances compared to the H line (longer) in IC/BPS patients compared with controls [61].

Bladder voiding in real-time was measured with MRI and computational fluid dynamics by calculating bladder wall displacement and urine flow dynamics, respectively, which is important for studying the effects of lower urinary tract symptoms (LUTS) [62]. This approach could be applied for IC patients, as bladder voiding is an important criteria assessment.

Multiregion MRI segmentation has been recently used to assess bladder cancer (BC) [63,64,65], which could be an interesting approach to assess regional changes in BWH, and should warrant future studies. As MRI has been extensively used to assess BC, there are possible lessons learned that could be applied to assess IC issues. For instance, multiparametric MRI is now being investigated to assess urinary BC, which includes DWI and/or DTI, dynamic contrast-enhanced (DCE) imaging, T2-weighted imaging, perfusion-weighted imaging (PWI), and measurement of T1 and T2 relaxation time constants [66,67,68,69,70,71,72,73,74,75,76,77,78,79]. As mentioned above, DWI and CE-MRI have already been considered for assessing bladder wall alterations associated with IC, however, adding changes in T1 and T2 relaxation values, as well as including T2-weighted imaging and PWI to the mix, may provide additional information on tissue structural damage (T1, T2, T2-weighted contrast) and vascular alterations (PWI).

Lastly, therapeutic applications of nanotechnology in BC are being considered to reformulate biological and cytotoxic agents for intravesical instillation, which would combine both diagnostic and therapeutic applications in one nanoformulation [80,81]. This approach could also be applied to assess a therapy for BWH, as well as measure the extent of BWH at the same time, where an MRI contrast agent with nanoparticles and an IC therapy, such as chondroitin sulfate or high molecular weight glycosaminoglycan (GAG), could be combined.

## 3. Conclusions

It is highly apparent that there are several diagnostic approaches that can be taken using MRI methodologies to assess either the bladder directly or brain regions associated with pelvic or bladder pain, and that ultimately these can be used to help assess therapeutic interventions for IC/BPS.

BWH can be assessed using CE-MRI, which would indicate the extent of bladder wall damage, duration of the damage, and when therapeutic interventions can be used to prevent the hyperpermeability. Preclinical evaluations of BWH biomarkers can also lead to clinical translation in the future. Bladder wall structural damage can also be assessed with DWI via the measurement of DW-MRI signal intensities or eventually ADC values. In the brain, DTI can be used to assess regional changes in white matter fiber tract alterations associated with pain. Pain associated with IC/BPS can also be assessed in the brains of patients via the use of fMRI. Both DTI and fMRI could possibly provide additional information on CNS remodeling associated with IC/BPS-associated pain and could identify possible therapeutics that could be effective with both bladder and brain pathologies. Regional cerebral blood flow can also provide some information of brain vascular alterations associated with IC/BPS. Lastly, morphological MRI can provide information on brain gray matter volumes or pelvic floor hypertonicity.

There are also multiparametric MRI approaches that are currently being considered for BC, which could be extended to assess bladder wall tissue structural changes, as well as monitor potential therapies.

The main disadvantage to using MRI routinely is expense. MRI can certainly provide information based on multiparametric methods, not only on the bladder itself (e.g., bladder wall structural changes with quantitative measurements regarding BWH with CE-MRI and ADC with DWI) but also regarding brain-related pathologies, which is not provided by other diagnostic tools, including cystoscopy and biopsy, and without the risks of the latter. This does not mean, however, that comparative studies between cystoscopy and MRI should not be done, as both imaging methodologies have their advantages and disadvantages. Biopsies should still be required to confirm histological changes, and future developments in image correlation technology could involve cystoscopy-guided-MRI-fused-biopsies in the future. Whether MRI might become the standard of care depends on how useful differentiating patients with BWH and CNS changes from those without them proves therapeutically.
